# Antisaccadic eye movements in middle-aged individuals with a family history of Alzheimer's disease

**DOI:** 10.3389/fnhum.2023.1143690

**Published:** 2023-04-20

**Authors:** Anjiao Peng, Wanlin Lai, Zhu Liu, Mingda Wang, Shujuan Chen, Xia Zhao, Yuanfeng Zhu, Lei Chen

**Affiliations:** ^1^Department of Neurology, Joint Research Institute of Altitude Health, West China Hospital, Sichuan University, Chengdu, Sichuan, China; ^2^Department of Clinical Research Management, West China Hospital, Sichuan University, Chengdu, Sichuan, China

**Keywords:** eye tracking, Alzheimer's disease, neurodegenerative diseases, cognition, antisaccade

## Abstract

**Background:**

Antisaccade is closely associated with cognitive ability in Alzheimer's disease (AD). However, studies regarding antisaccade in the early stages of AD are scarce. Considering that first-degree family history is a well-established risk factor for AD, we explored the influence of family history on the performance of antisaccade tasks in individuals with normal cognition.

**Methods:**

In total, 44 participants (aged 50–66 years) with a family history of AD (FH+) and 44 age-, gender-, and educational level-matched controls (FH-) were enrolled in our study. After cognitive assessment using the Montreal Cognitive Assessment and Mini-mental State Examination, participants underwent antisaccade trials, and all parameters were recorded using an eye tracker.

**Results:**

While the average velocity was relatively lower in FH+ individuals than in FH− individuals (107.9 ± 14.3°/s vs. 132.9 ± 23.7°/s, *p* < 0.001), FH+ individuals surprisingly showed relatively fewer uninhibited reflexive saccades (44.7 ± 26.0% vs. 56.2 ± 24.7%, *p* = 0.037) than the control group. They also required a relatively shorter time to detect and correct false saccades (121.6 ± 40.7 ms vs. 143.9 ± 37.0 ms, *p* = 0.023).

**Conclusions:**

This study showed that family history is associated with alterations in antisaccadic parameters, suggesting that eye tracking can be used to assess oculomotor control and executive function in individuals at risk of developing dementia.

## Introduction

Alzheimer's disease (AD) is the leading cause of dementia, which has been estimated to affect 131.5 million people worldwide by 2050 (Sengoku, 2020). Several tests, including functional magnetic resonance imaging (MRI), positron emission tomography, and cerebrospinal fluid-based biological markers, such as amyloid beta, provide us with important information about the structural and metabolic alterations in AD. However, these tests do not directly reflect deteriorating cognitive processes.

Saccades are defined as rapid eye movements that jump from one point to another and involve a wide range of cortical and subcortical networks. It has been used to evaluate perception, attention, and cognition in patients with AD for many years (Peltsch et al., 2014; Holden et al., 2018; Chehrehnegar et al., 2022; Opwonya et al., 2022). An antisaccade task requires individuals to inhibit the production of a visually guided saccade and produce an antisaccade, which is a saccade to a mirror location in space where there is no visual stimulus. This task has been found to be sensitive enough to be effective in detecting cognitive decline. Generally, patients with AD tend to exhibit a greater number of errors and longer latency in the antisaccade task (Yang et al., 2013; Peltsch et al., 2014; Opwonya et al., 2022). Patients with mild cognitive impairment (MCI) performed similarly to those of patients with AD (Yang et al., 2013; Peltsch et al., 2014; Laurens et al., 2019; Chehrehnegar et al., 2022) indicating that antisaccadic parameters are promising biomarkers for detecting dementia (Nie et al., 2020). Before the occurrence of clinical symptoms, individuals with AD often experience 20 to 30 years of prodromal stage (Jansen et al., 2015). A critical stage in exploring the early pathogenesis of AD. Therefore, it is important to study antisaccadic eye movements at this stage. To the best of our knowledge, studies in this field are limited.

First-degree family history is a well-established risk factor for AD (Talboom et al., 2019; Manzali et al., 2021), which increases the risk of both heritable and non-heritable forms of the disease (Yi et al., 2018). Scale-based studies have found that positive family history is associated with cognitive changes in different age groups, including children, middle-aged people, and older adults (Bloss et al., 2008; Zeng et al., 2013; Aschenbrenner et al., 2016). In this study, we aimed to explore the effect of positive family history on the performance of saccades in middle-aged, cognitively normal individuals.

## Methods

### Participants

In total, 44 participants, aged 50 to 66 years, with a history of AD in their father, mother, or both, and another 44 age-, gender-, and education-matched controls were enrolled from the Natural Population Cohort Study of the West China Hospital at Sichuan University (Peng et al., 2022). The control group consisted of individuals whose parents or siblings had not been diagnosed with AD. The exclusion criteria were as follows: AD, Parkinson's disease, dementia with Lewy bodies, traumatic brain injury, stroke, depression, anxiety, bipolar disorder, substance abuse, ophthalmological or auditory impairment, and a history of using medicines that can improve cognition. This study was approved by the ethics committee of the West China Hospital, Sichuan University, and informed consent was obtained from all the participants.

### Basic information

The participant demographics (age, gender, marital status, educational level, and occupation) were extracted from the baseline dataset of the cohort study. Marital status was categorized as married or unmarried (divorced, separated, or widowed). Educational levels included primary (< 6 years of education), middle (6–9 years of education), high (9–12 years of education), and advanced education (> 12 years of education). Occupations were categorized as employed, unemployed, or retired. All participants underwent general cognitive assessments using the Montreal Cognitive Assessment (MoCA) (Nasreddine et al., 2005). and Mini-mental State Examination (MMSE) (Li et al., 2016).

### Experimental set-up

The participants sat in a dark room with their heads restrained on a chin rest. Eye position was recorded using an infrared video-based eye tracker (EyeMind 2000; Jasmine Science and Technology Ltd., Chengdu, China; the experimental setup is displayed in the Supplementary material). The sampling rate and spatial accuracy of this eye tracker were 1 kHz and ≤ 0.025°. All visual stimuli were displayed on a 27-inch LCD monitor (1,920 × 1,080 resolution, 100 Hz refresh rate) positioned 57 cm in front of the participants. Visual stimuli presentation and behavioral data collection were controlled using MATLAB (R2009b; MathWorks, Natick, MA, USA) with Psychtoolbox (PTB-3) running on a Windows PC system (HP).

All participants underwent antisaccade trials. During the tests, the participants were instructed to fixate on the cross at the center of the screen and then to look in the opposite direction of the target at an equal distance from the center as quickly as possible when the central cross disappeared, and the peripheral white spot appeared simultaneously. Specifically, the trial began with the appearance of a fixation point (a white cross, horizontal 1° × vertical 1° in size) at the center of the screen. The participants were required to look at it as soon as it appeared. If the participants' eyes entered a check window (4° in radius) and remained within the window for 800 ms, the fixation point disappeared. A visual cue (a white dot, diameter 1° in size) simultaneously appeared randomly at one of the four peripheral locations (left, right, up, and down; at an eccentricity of 10°). The participants were instructed to make a saccade in the direction opposite to the visual cue (mirror location) as accurately and rapidly as possible. The visual cue disappeared after the eyes entered and stayed within a check window (4° in radius) for 800 ms. If the participants' eyes did not enter the fixation window 1,000 ms after fixation point onset or did not make a saccadic eye movement 2,000 ms after the visual cue onset, the trial was aborted. At the end of each trial, there was an 800-ms intertrial interval during which a blank screen appeared. Each participant completed 12 experience trials to ensure that they understood the task and then completed 40 formal trials.

### Eye-movement signal processing

Before saccade detection, a data-smoothing procedure using a sliding window was performed to minimize the influence of noise produced during the recording period. Subsequently, artifacts, such as blinks, were removed using flags marked by the eye tracker during data recording. Subsequently, autovelocity threshold saccade detection was performed. The saccade-detection procedure consisted of three steps. The first step was to identify all the potential saccades in each trial. Potential saccades were detected based on the criteria of absolute velocity >15°/s and duration >10 ms. The whole-eye trace was divided into several different segments by the potential saccades. Each segment consisted of two parts: one part of the eye trace before the saccade onset and the other part during the saccade. The second step was to calculate the velocity threshold for each segment based on the segment part before the saccade onset. The velocity criteria were set as mean ± 2.58 times the standard deviation (SD) (99% confidence interval). The third step was to redetect the saccade onset and offset based on the new velocity threshold from the second step for each segment. There was no amplitude or acceleration threshold for initial saccade detection, but microsaccades (amplitude < 1°) were removed for later data analysis.

All raw eye-tracking features were extracted from the eye-tracker software. The following saccadic parameters of each task were used for further analyses: (1) uninhibited reflexive saccades (i.e., participants made saccades to the location of visual cues in the wrong direction); (2) mean saccade latency (i.e., the mean reaction time for correct antisaccades only); (3) average velocity; and (4) the intersaccade interval (i.e., the interval between uninhibited reflexive saccades and saccades toward the target).

### Statistical analysis

Continuous variables were presented as mean and SD and calculated using a *t*-test when values were normally distributed and conformed to a normal distribution. Otherwise, variables were presented as medians and interquartile ranges and calculated using the Mann–Whitney *U*-test. Categorical variables were described as frequencies and percentages and compared using the chi-square test or Fisher's exact test. All statistical analyses were performed using Python software (version 3.8.3) and the package sciPy (version 1.5.3), and the significance level was set at 5% two-sided.

## Results

### Participant characteristics

A total of 44 participants with a family history of dementia (FH+) and 44 age-, gender-, and education-matched individuals (FH-) were included. In the FH+ group, 25 (56.8%) participants had a maternal history of AD, 17 (38.6%) had a paternal history of AD, and 2 (4.6%) had both parents diagnosed with AD. The mean age was 56.2 ± 3.9 years, and the women accounted for the majority of the participants in both groups (37, 84.1%). The global cognitive performance measured using the MMSE (27.5 ± 2.3 vs. 27.0 ± 2.1, *p* = 0.36) and MoCA (24.8 ± 3.8 vs. 23.5 ± 3.3, *p* = 0.094) was comparable between the two groups. Moreover, the two groups were comparable in terms of marital status and educational level (Table 1).

**Table 1:** Demographic features for participants with or without family history of Alzheimer's disease.

	**FH+ (*n =* 44)**	**FH- (*n =* 44)**	***p*-value**
Age (mean ± SD, years)	56.2 ± 3.9	56.2 ± 3.9	1.0
Gender (female, %)	37 (84.1)	37 (84.1)	1.0
**Family history**			
Maternal	25 (56.8)	-	
Paternal	17 (38.6)	-	
Parental	2 (4.6)	-	
Educational level (%)			1.0
Primary school	8 (18.2)	8 (18.2)	
Middle school	20 (45.5)	20 (45.5)	
High school	12 (27.3)	12 (27.3)	
Advanced	4 (9.1)	4 (9.1)	
Marital status (married, %)	40 (91.0)	37 (84.1)	0.33
Occupation (%)			0.89
Employed	9 (20.5)	9 (20.5)	
Retired	20 (45.5)	22 (50.0)	
Unemployed	15 (34.1)	13 (29.6)	
MoCA (mean ± SD)	24.8 ± 3.8	23.5 ± 3.3	0.094
MMSE (mean ± SD)	27.5 ± 2.3	27.0 ± 2.1	0.36

FH, Family history of Alzheimer's disease; MMSE, Minimental State Examination; MoCA, Montreal Cognitive Assessment; SD, standard deviation.

### The saccadic performance of individuals with or without a family history

**Figure 1:**
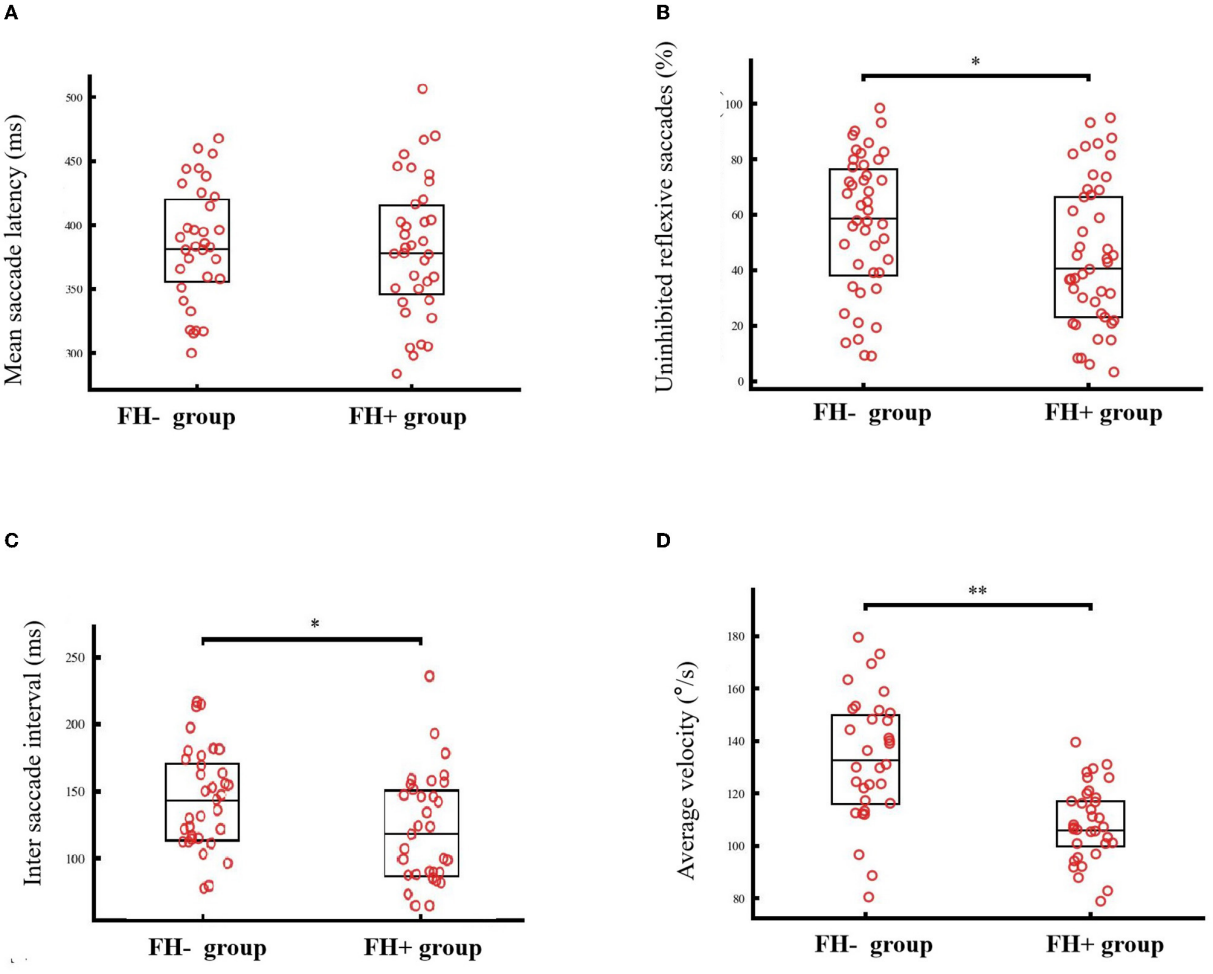
The saccadic performance of individuals with (FH+) or without family history (FH-). No group difference was observed regarding the mean saccade latency [380.8 ± 53.8 ms vs. 383.5 ± 45.9 ms, *T*_67_ = *0.227, p* = 0.82, **(A)**], but FH+ group showed relatively fewer uninhibited reflexive saccades [44.7 vs. 56.2%, *p* = 0.037, **(B)**], shorter saccade interval between uninhibited reflexive saccade and saccade toward the target [121.6 ms vs. 143.9 ms, *p* = 0.023, **(C)**]. While, the FH+ group displayed relatively lower average velocity than the FH- group [107.9°/s vs. 132.9°/s, *p* < 0.001 **(D)**]. ^*^p < 0.05, ^**^p < 0.001.

The FH+ group presented significantly fewer uninhibited reflexive saccades (44.7 ± 26.0% vs. 56.2 ± 24.7%, *T*_86 =_ *2.120, p* = 0.037). No group difference was observed regarding the mean saccade latency (380.8 ± 53.8 ms vs. 383.5 ± 45.9 ms, *T*_67 =_ *0.227, p* = 0.82), but further analyses showed that the FH+ group had a relatively shorter saccade interval between uninhibited reflexive saccade and saccade toward the target (121.6 ± 40.7 ms vs. 143.9 ± 37.0 ms, *T*_62 =_ *2.319, p* = 0.023). These results suggest that the FH+ group required a relatively shorter time to detect and correct false saccades. The results of the average velocity showed the opposite trend with the FH+ group, displaying a relatively lower average velocity than the FH- group (107.9 ± 14.3°/s vs. 132.9 ± 23.7°/s, *T*_50_ = *5.149, p* < 0.001) (Figure 1).

## Discussion

We hypothesized that cognitively healthy middle-aged individuals with a positive family history of AD who have normal cognitive function would show impaired performance on antisaccade tasks, as has been observed in patients with AD or MCI (Yang et al., 2013; Peltsch et al., 2014; Holden et al., 2018; Oyama et al., 2019; Chehrehnegar et al., 2022; Opwonya et al., 2022). Unexpectedly, we found that these individuals showed fewer uninhabited reflexive saccades and needed less time to correct them, reflecting better executive and cognitive abilities.

Similar to previous findings in patients with AD and (Yang et al., 2011; Peltsch et al., 2014; Chehrehnegar et al., 2019) mild cognitive decline (Peltsch et al., 2014; Laurens et al., 2019; Chehrehnegar et al., 2022) and young apolipoprotein E (APOE) 4 carriers (Niechwiej-Szwedo et al., 2018), we found that individuals with a family history of AD showed a relatively lower velocity. Considering that velocity is a measure of the integrity of motor processes leading to saccade generation, which involve a large range of oculomotor control areas, such as the occipital (Shakespeare et al., 2015), frontal, and parietal cortex (Bisley et al., 2011), cerebellum, and superior colliculus (Munoz and Coe, 2011), the relatively lower velocity in individuals with a positive family history may reflect impaired function involving these eye movement control areas.

Unexpectedly, the error rate was relatively low among individuals with a positive family history. During the antisaccade task, individuals need to look directly at the mirror position of the target, which involves complex progress in attention shifting and decision-making and is sensitive to evaluating executive and cognitive abilities (Li et al., 2016; Chehrehnegar et al., 2022). The error rate during antisaccade tasks was thus found to correlate well with executive function and has been proven to be a sensitive biomarker of AD and MCI (Peltsch et al., 2014; Laurens et al., 2019; Chehrehnegar et al., 2022). Structurally, the error rate in the antisaccade task is associated with the frontal eye fields and frontoparietal cortex (Heuer et al., 2013). The relatively lower error rate in individuals with a family history may reflect greater activation of these areas. Our findings that individuals with a positive family history presented significantly fewer uninhibited reflexive saccades and used a relatively shorter time to detect and correct false saccades also reflected better cognition in these individuals.

Hitherto, compelling evidence has suggested that the disease course of AD is more likely to be an inverted U-shaped pattern than a continuous decline, meaning that people may experience a better functional stage before the decline of cognitive ability (Corriveau-Lecavalier et al., 2020). Findings from functional MRI-based studies revealed that individuals with an early stage of dementia or who were cognitively normal but at a higher risk of developing dementia (carrying the APOE4 allele or having a family history of dementia) presented with greater brain activation, supporting this inverse quadratic hyperactivation hypothesis (Bookheimer et al., 2000; Burggren et al., 2002; Bondi et al., 2005; Wierenga et al., 2013; Green et al., 2023). These altered hyperactivations in MRI may present corresponding functional changes and better performance during antisaccade trials. Our results suggest that the inverted U-shaped hypothesis can be further verified in prospectively designed studies using an antisaccade task. In contrast, social factors related to family history status may also contribute to these results. When a person's father or mother has AD, they may develop a deeper understanding of the burden of dementia. Knowledge of a higher risk of developing AD may motivate them to change their lifestyle, which could potentially improve their cognition at a certain stage. However, these hypotheses must be verified in future studies.

## Limitations

This study had some limitations. First, owing to the nature of the cross-sectional design, we could not assess the dynamic impact of family history on saccadic performance in different age groups. Second, although family history is a well-established risk factor for AD, other potential risk factors may also influence the performance of saccade tasks. Finally, this study included a relatively small number of participants. Thus, these results need to be further confirmed in future studies with larger sample sizes.

## Conclusion

The results of this study indicate that eye tracking can be used to assess oculomotor control and executive function in individuals who are at risk of developing dementia.

## Data availability statement

The raw data supporting the conclusions of this article will be made available by the authors, without undue reservation.

## Ethics statement

The studies involving human participants were reviewed and approved by the Ethics Committee of West China Hospital, Sichuan University. The patients/participants provided their written informed consent to participate in this study.

## Author contributions

Full access to all the data in the study, takes responsibility for the integrity of the data, and the accuracy of the data analyses: LC. Study concept, design, data analyses, interpretation, and critical manuscript revision for important intellectual content: AP, WL, and LC. Data acquisition: AP, ZL, MW, SC, XZ, and YZ. All authors contributed to the article and approved the submitted version.
