# Supplementary figures and images for: Antisaccadic eye movements in middle-aged individuals with a family history of Alzheimer's disease
Source: Front Hum Neurosci. 2023 Apr 20;17:1143690. doi: 10.3389/fnhum.2023.1143690 (PMC10157194; doi:10.3389/fnhum.2023.1143690)

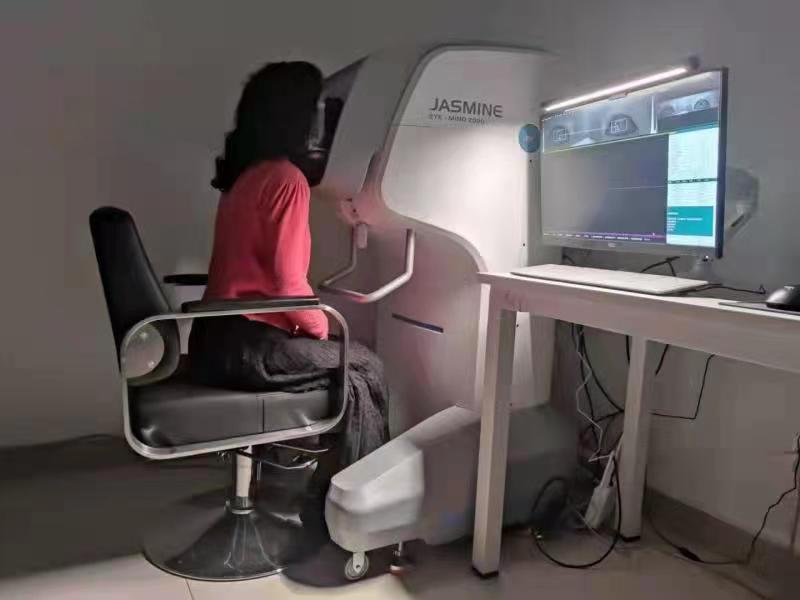


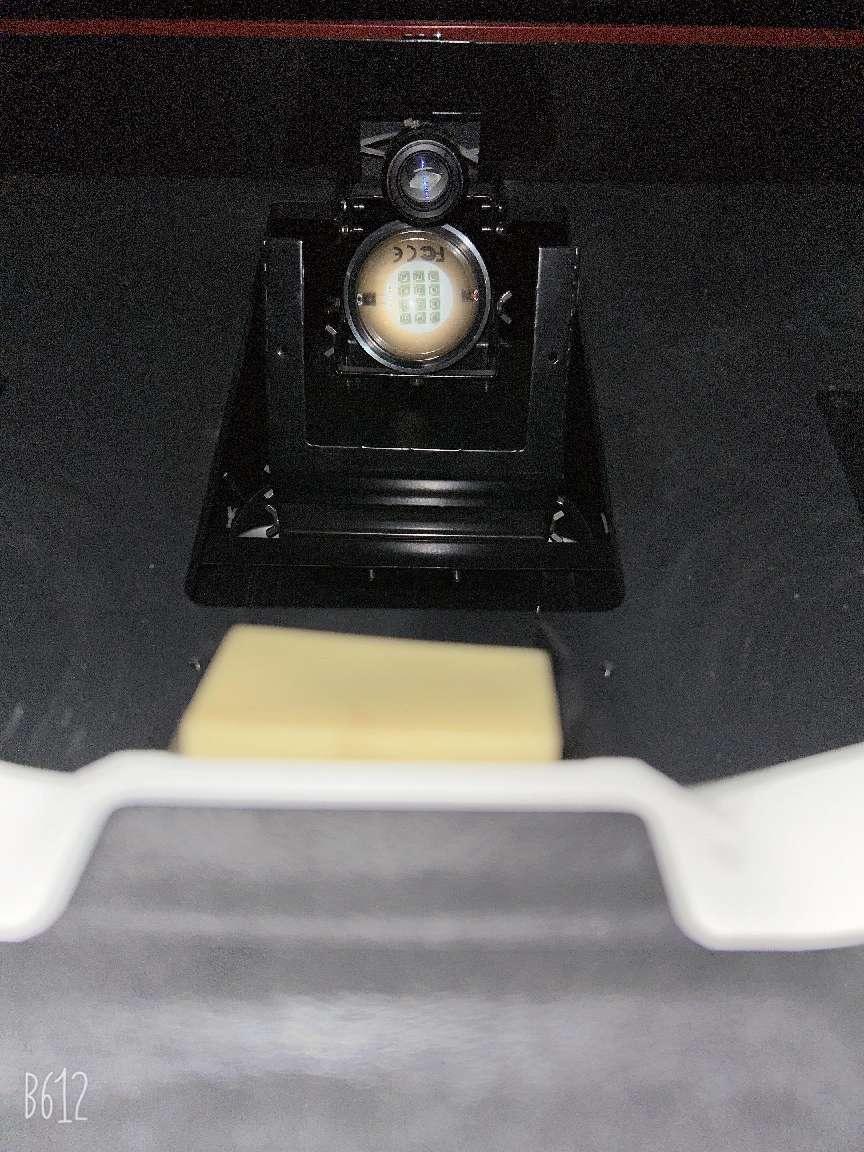


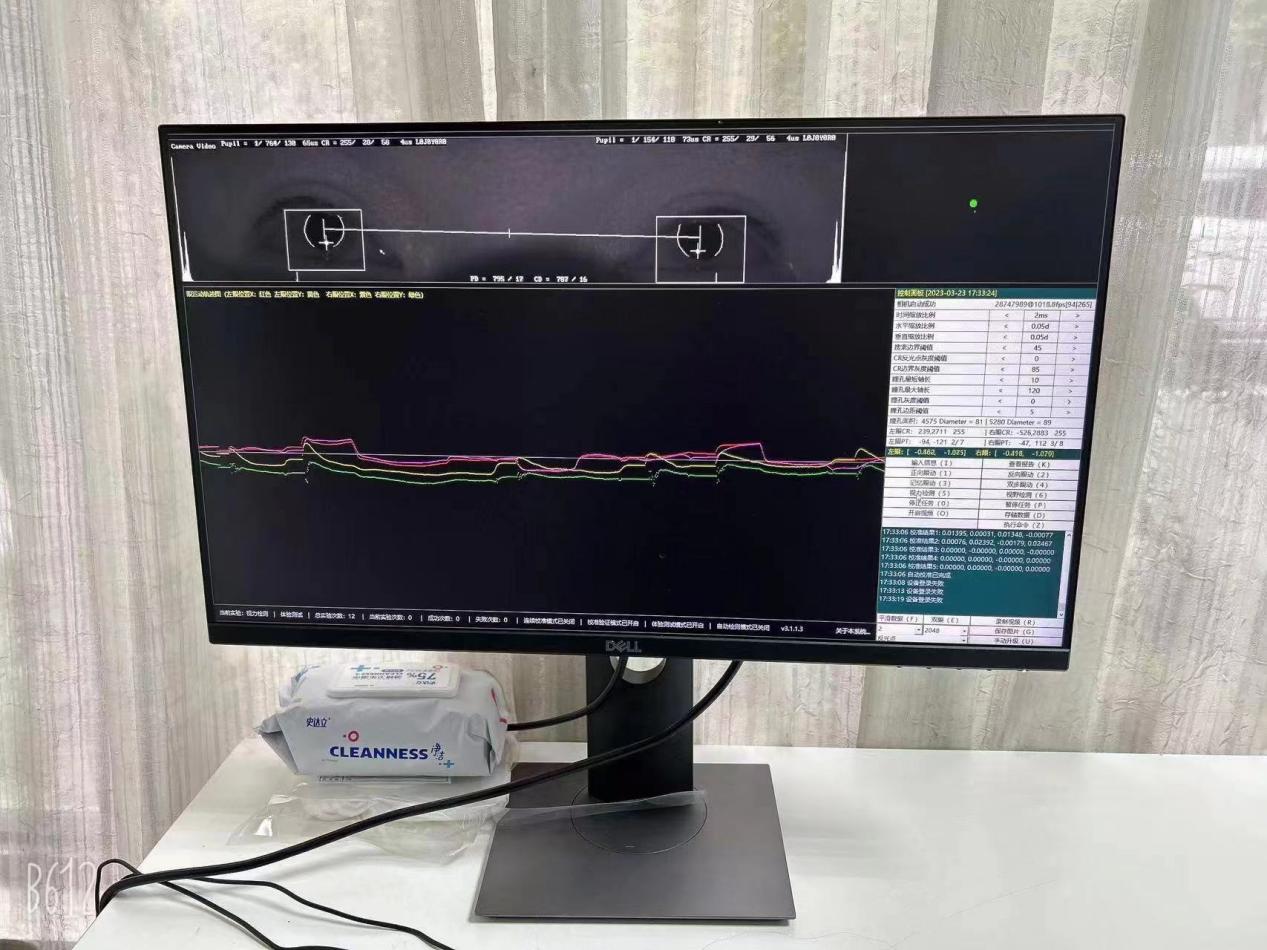


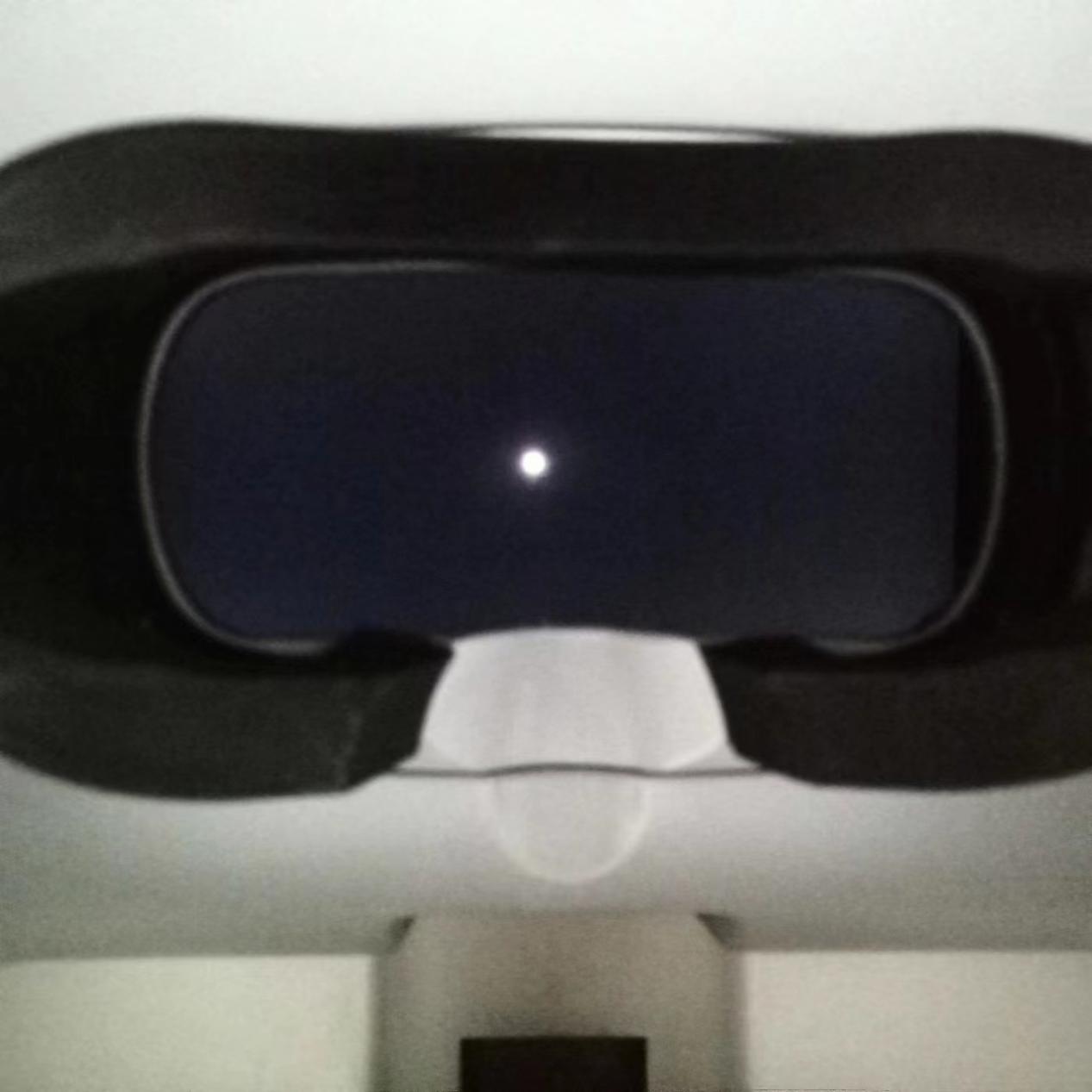

Supplement: Supplementary file 1 [file Data_Sheet_1.docx]
